# Beyond the Genomic Mutation: Rethinking the Molecular Biomarkers of K-RAS Dependency in Pancreatic Cancers

**DOI:** 10.3390/ijms21145023

**Published:** 2020-07-16

**Authors:** Carla Mottini, Luca Cardone

**Affiliations:** 1 Department of Research, Advanced Diagnostics and Technological Innovations, IRCCS Regina Elena National Cancer Institute, 00144 Rome, Italy; carla.mottini@ifo.gov.it; 2 Institute of Biochemistry and Cellular Biology, CNR National Research Council, 00015 Rome, Italy

**Keywords:** pancreatic cancer, *K-RAS* oncogene, oncogene dependency, targeted therapies, biomarkers, genomic mutations, transcriptomics, metabolomics

## Abstract

Oncogenic v-Ki-ras2 Kirsten rat sarcoma viral oncogene homolog (K-RAS) plays a key role in the development and maintenance of pancreatic ductal adenocarcinoma (PDAC). The targeting of K-RAS would be beneficial to treat tumors whose growth depends on active K-RAS. The analysis of *K-RAS* genomic mutations is a clinical routine; however, an emerging question is whether the mutational status is able to identify tumors effectively dependent on K-RAS for tailoring targeted therapies. With the emergence of novel K-RAS inhibitors in clinical settings, this question is relevant. Several studies support the notion that the K-RAS mutation is not a sufficient biomarker deciphering the effective dependency of the tumor. Transcriptomic and metabolomic profiles of tumors, while revealing K-RAS signaling complexity and K-RAS-driven molecular pathways crucial for PDAC growth, are opening the opportunity to specifically identify K-RAS-dependent- or K-RAS-independent tumor subtypes by using novel molecular biomarkers. This would help tumor selection aimed at tailoring therapies against K-RAS. In this review, we will present studies about how the K-RAS mutation can also be interpreted in a state of K-RAS dependency, for which it is possible to identify specific K-RAS-driven molecular biomarkers in certain PDAC subtypes, beyond the genomic K-RAS mutational status.

## 1. Oncogenic K-RAS: A Critical Driver for Pancreatic Cancer

Pancreatic ductal adenocarcinoma (PDAC) is a major cause of cancer-related death with an overall five-year survival rate of only 8% [1,2]. PDAC is diagnosed at an advanced, inoperable stage in the vast majority of cases and most of the patients diagnosed with surgically resectable disease recur within the first 2–3 years after the operation [3]. Current systemic first-line treatment for advanced inoperable PDAC includes polychemotherapy regimens such as folinic acid/ 5-fluorouracil/irinotecan/oxaliplatin,(FOLFIRINOX),cisplatin/nab-paclitaxel/capecitabine/gemcitabine (PAXG), gemcitabine/nab-paclitaxel, and gemcitabine monotherapy in a small sub-group of elderly, frail, or unfit patients. Primary chemoresist/ance or recurrence rates in PDAC remain high, and overall survival from the start of first-line ranges approximately from 8 to 12 months [4,5,6]. Currently, no validated prognostic or predictive biomarkers exist for PDAC, except for general clinical criteria (performance status, disease burden, CA19.9 levels), and no targeted or immune-based therapies have proven to be effective so far, although a large number of clinical trials are ongoing and efficacy data for novel treatments are awaited [7,8,9].

The RAS pathway is one of the most frequently altered pathways in cancer, found in approximately 19% of all human cancer harboring *RAS* gene mutations [10]. Among the three major isoforms of oncogenic *RAS*, *K-RAS* is the most frequently mutated [11,12,13]. Mutation of *K-RAS* is the initiating genetic event of pancreatic intraepithelial neoplasias (PanINs) and is required to drive PDAC development and tumor maintenance [14,15,16,17,18] Oncogenic mutant *K-RAS* is found in about 88% of PDAC [10]. Oncogenic mutation in K-RAS protein leads to aberrant or constitutive signaling even in the absence of growth factors, leading to increased proliferation, invasion, and metastasis [19]. Inactivating mutations in crucially tumor suppressor genes, particularly *CDKN2A/p16*, *TP53*, and *SMAD4*, cooperate with oncogenic *K-RAS* to promote aggressive PDAC tumor growth and metastasis [19,20,21,22,23,24,25,26].

K-RAS is a member of the RAS family of Guanosine Tri-Phosphate(GTP)-ases that regulates several cellular processes including survival, proliferation, differentiation, migration, and apoptosis [27]. RAS proteins function as molecular switches promoting conversion from an inactive to an active GTP-bound state. Though tightly controlled in normal cells, the mutation in K-RAS gene leads to constitutive GTP-bound K-RAS, rendering constitutively activated RAS protein and determining the persistent activation of downstream signaling pathways resulting in uncontrolled activation of proliferation and survival pathways [28,29,30,31]. The mutations in K-RAS consist of single amino acid substitutions and are predominant at residues G12, G13, and Q61. Oncogenic mutations of G12 or G13 create a steric block that prevents the hydrolysis of GTP, whereas substitutions of Q61 interfere with the coordination of a water molecule required for GTP hydrolysis; these point mutations lead to a prevalence of the GTP-bound state and to the constitutive activation of K-RAS [19].

Once in its active form, K-RAS engages complex and dynamic downstream effectors such as the RAF/MEK and the phosphatidylinositol 3-kinase (PI3K)/AKT pathway. The Mitogen-Activated Protein Kinase (MAPK) pathway is a key mediator of oncogenic K-RAS signaling and BRAF is the principal mediator of MAPK signaling in K-RAS dependent cancer growth. The BRAF V600E mutations are mutually exclusive with K-RAS mutations [32]. However, genetic studies in mice models revealed that *BRAF ^V600E^* mutation is sufficient to induce PanIN formation in the pancreas of *K-RAS* wild-type (WT) mice, and to develop lethal PDAC when combined with a *TP53* mutation [33]. The PI3K-dependent pathway drives tumor growth and cooperates with oncogenic K-RAS to develop PDAC [34,35]. The major driver mutations in this pathway that promote pancreatic tumor development include mutations in the catalytic and regulatory PI3K subunit, amplification of the PI3K downstream effector AKT2, and deletion/loss of tumor suppressor Phosphatase/TENsin homolog deleted on chromosome 10 (PTEN), a negative regulator of PI3K/AKT signaling [36,37,38].

It is important to mention that a relatively large proportion of patients with PDAC display germline mutations of some DNA damage repair (DDR) genes. Specifically, 18% of PDAC harbor mutations in homologous recombination (HR) DDR pathways such as *BRCA1* and *BRCA2* [39], and the BRCA2 inactivation in combination with p53 deficiency promotes K-RAS driven PDAC development [40,41].

It goes without saying that the genomic landscape of PDAC shows multiple genetic events, most of them contributing to tumor maintenance in cooperation with the K-RAS activation, most likely with a different degree of dependency according to the history of the tumor development, staging, or treatments. Deciphering the effective dependency of the tumor on K-RAS or on alternative oncogenes is key to promote targeted therapies in PDAC.

## 2. Defining the K-RAS Dependency in PDAC

*K-RAS* mutation represents a common genetic event in PDAC, being mutated in almost 88% of cases [10,42]. However, contrary to preclinical studies, clinical approaches have demonstrated poor efficacy of treatments targeting the K-RAS pathway in PDAC tumors carrying a K-RAS mutation. One of the potential explanations is the possibility that the genomic *K-RAS* mutation is not an efficacious molecular determinant for tumor dependency on K-RAS activation. Indeed, the absence of K-RAS gene mutations does not always correlate with K-RAS pathway inactivity due to the activation of the other components of the network [43,44], and conversely, the presence of *RAS* mutations does not necessarily predict for dependency. This can depend on the activation of additional active molecular pathways that can complement or subside for K-RAS activation. Thus, determining the genomic mutational status of specific genes is not always beneficial for predicting pathway activation and the drug response with targeted compounds [45].

Assessing K-RAS pathway activation status by more comprehensive methods will help better predict the K-RAS dependency of tumors. Years have passed since the concept of oncogene addiction was first proposed, linking single dominant oncogene to tumor growth and survival [46]. Omics studies, such as genomics, transcriptomics, and metabolomics can lead to extensive molecular profiles, which act as tools to reevaluate the traditional definition of addiction and oncogene dependency as a functional definition based on the oncogene-driven phenotype, regardless of the presence or not of a specific oncogenic gene mutation. A large number of observations in animal models and pancreatic cancer cell lines revealed that the *K-RAS* gene, although mutated or overexpressed, is dispensable in a subset of human and mouse K-RAS mutant PDAC cell lines. By using RNA interference, inducible transgenic models or Clustered Regularly Interspaced Short Palindromic Repeats (CRISPR)-Cas9 technology, it has been possible to classify two subtypes of PDACs harboring the K-RAS mutation: tumors in which a K-RAS depletion led to apoptosis and thus they are considered as “K-RAS-dependent” and others that are resistant to K-RAS depletion, without a sign of apoptosis, and considered as “K-RAS-independent” [47,48,49,50,51,52,53]. The extensive molecular characterization of such models shed a light on additional features that would be missed based on simple genomic classification of the tumor, with the potential of a profound implication from a therapeutic and prognostic point of view.

The goal of this review is to provide an overview of emerging molecular markers of K-RAS oncogene dependency, regardless of the genomic mutation status. Gene expression profile studies, in particular, allow to understand if the K-RAS pathway could be activated by mutations of the *K-RAS* gene or by many other mechanisms, and they help to deconstruct the K-RAS network contribution in tumor progression [45,48,54]. In addition, metabolomics studies identified pathways and metabolites that are specifically enriched in K-RAS-dependent PDAC to mediate a metabolic reprogramming relevant to tumor growth. Thus, multiple and specific molecular biomarkers underlining the oncogenic phenotype associated with a real dependency on K-RAS oncogene in PDAC are emerging. The translational value of such information is manifold since i) it helps to find novel diagnostic biomarkers that could overcome the limitation of a genomic-based approach for an effective determination of K-RAS dependency and ii) it provides the ground for novel therapeutic strategies to define effective targeted therapy against a subclass of PDAC patients, whose tumors have K-RAS dependency and actionable vulnerabilities.

In the next paragraphs, we will discuss molecular profiling based on transcriptomic and metabolomics studies that provided novel markers for K-RAS dependency in PDAC.

## 3. Scores of K-RAS Dependency Based on Gene Expression Signatures

Specific gene expression signatures can be associated with oncogenic mutations and deregulated signaling pathways in tumors [45,55]. Several studies demonstrated the potential of using gene expression profiles of cancer cells to analyze oncogenic pathways [45,56]. Gene expression signature is also a powerful tool for predicting drug response in vitro linked to specific molecular pathways [45,57,58]. In tumor samples, gene expression signature can reveal molecular pathways that are activated independently from mutation status, confirming that mutations were no obvious predictors for pathway activation [44]. As an example, in bladder urothelial carcinoma, Epidermal Growth Factor Receptor (EGFR)-, K-RAS-, and RAF-dependent pathways were activated in 42%, 22%, and 38% of cases, but only three patients carried the *EGFR* mutation and no mutation in *K-RAS* or *RAF* was found. Furthermore, reverse phase protein array (RPPA) demonstrated significant MAPK pathway activity in both K-RAS-mutant lung adenocarcinoma and *K-RAS* wild-type samples [44].

Oncogenic K-RAS-specific signatures have been derived from cancer models [48,51,59]. Gene expression profiling studies in some cell lines and human tumors have allowed identifying a more comprehensive K-RAS-dependent network able to understand the pathway activation’s status and to better describe the K-RAS-dependent molecular network [47,48]. In their studies, Singh et al. [47] stratified K-RAS mutant pancreatic and lung cancer cell lines into K-RAS-dependent or K-RAS-independent subtypes, according to the ability of K-RAS to support cell viability. Specifically, the ablation of mutant *K-RAS* by RNAi affected cell viability and induced apoptosis with differential sensitivity between cell lines. A K-RAS dependency index, in a subset of pancreatic and lung cancer cell lines harboring oncogenic *K-RAS*, was defined based on caspase-3 cleavage values. To better delineate the molecular mechanisms that distinguish K-RAS-dependent and independent cell lines, a gene expression signature identifying genes differentially expressed in these two groups was derived. The authors identified a 250 gene-based gene signature to define dependency in PDAC (Table 1).

With a different approach and models, Loboda et al. identified a 147 gene-based *K-RAS* gene signature [48] that was associated with tumors with a *K-RAS* dependency (Table 1). By using a K-RAS knockdown-based strategy, the authors demonstrated that in a panel of lung and breast tumor cell lines, not all K-RAS mutated cells were dependent on K-RAS signaling, and some cells carrying wild-type *K-RAS* exhibited a *K-RAS* dependency. Furthermore, the K-RAS dependency gene signature was highly correlated with MEK and ERK phosphorylation and with the cellular response after a MEK inhibitor treatment, suggesting the clinical relevance of such gene signature to predict the response to K-RAS pathway inhibitors [48].

The potential translational value of these scores was validated by Mottini et al. [54] who showed that the gene expression signatures identified by Loboda and Singh showed a high correlation among them to classify K-RAS-dependent or K-RAS-independent PDAC cell lines according to signature similarity scores. In addition, the authors demonstrated, for the first time, that both genetic signatures derived by Loboda and Singh were able to identify a dependency on K-RAS in patient-derived xenograft (PDX) models of PDAC, which is a more reliable, patient-like experimental system, demonstrating the predictive capability of these two gene signatures identified by in-vitro cancer models. The authors also reported an in-vivo discrepancy between the two K-RAS-dependent gene signatures; they argued that the discrepancy could reflect the different approaches used to derive such signatures and potentially be associated with differences in how the microenvironment can influence gene signatures, in particular, the one identified by Singh et al. [47]. Indeed, classification of PDAC-PDXs using the ones identified by Singh better match the molecular subtypes recently identified in PDAC patients by applying tumor-specific factors [60,61]. On the other hand, the signature identified by Loboda might be associated with a higher degree of cell-autonomous signature, which is potentially less influenced by microenvironmental components. The authors concluded that, based on the evidence of PDX data and The Cancer Genome Atlas (TCGA) data, using combined molecular signatures might be a robust predictive tool to infer oncogene dependency from tumor biopsy or PDX models.

In an attempt to identify the mechanism for K-RAS independency in PDAC, Kapoor and colleagues [50] discovered that, upon K-RAS suppression, about half of the tumors relapsed thanks to activation of a transcriptional program controlled by the cooperation of the Yes-Associated Protein 1/TEA Domain Transcription Factor 2 (YAP1/TEAD2) transcription factors complex to promote cell cycle, DNA replication, and tumor maintenance in the absence of oncogenic K-RAS. Transcriptomic and network analysis profiles in the K-RAS-independent YAP1-driven tumors provided substantial evidence on how YAP activation bypasses K-RAS mutations by supporting the transcriptional pathways that are key K-RAS targets. Thus, YAP1 supports pathways activated by mutated K-RAS, and active YAP1 pathway emerged as a putative marker and therapeutic target against pancreatic cancers showing K-RAS independency [50].

It is worth noting that the above-cited studies indicate that, in certain cell lines, the definition of K-RAS dependency might suffer a discrepancy, due to the type of in vitro biological assay. This likely depends on cell-specific genetic mutations and molecular pathways activations, within the K-RAS network, that are engaged under the relevant biological assay. For example, growing cells into two-dimensional (2D) or 3D cultures, or instead, a clonogenic assay, might differentially engage K-RAS activation, generating differences and discrepancy for the K-RAS dependency outcome in the same cell line. Such discrepancy indicates the need for an accurate and robust validation for biomarkers of dependency, and it suggests that, besides in vitro-based studies, further validation of dependency biomarkers in a more physiological context such as in vivo tumor models is required. Moreover, the implementation of multiple transcriptional molecular scores for dependency would be of advantage for an accurate definition of *K-RAS* dependency, in particular when this information aims to predict drug response [54].

Transcriptomic approaches have also been instrumental to different molecular classifications of pancreatic ductal adenocarcinoma subtypes. By examining expression data from human and mouse cell lines, Collisson et al. classified PDAC into three subtypes termed "classical", "quasi mesenchymal", and exocrine-like [49]. Notably, the classical subtype was defined by high expression of adhesion specific and epithelial genes and was reported to confer the best chance of survival. On the other hand, the quasi-mesenchymal subtype showed a higher expression of mesenchymal associated genes with a poorly differentiated phenotype and related to a poor prognosis. Bailey et al. [62] analyzed transcriptomic data from tumor tissues containing the tumor microenvironment and identified a new “immunogenic” subgroup associated with immune stroma cell populations. Expression analysis defined four subtypes as “squamous”, “pancreatic progenitor”, “immunogenic”, and “aberrantly differentiated endocrine exocrine”. The squamous subtype showed the worst overall survival and overlaps with the quasi-mesenchymal tumor subtype defined by Collison et al. [49]. Tumors of the squamous subtype were reported to be associated by the presence of gene expression programs and regulatory networks involved in the inflammatory response, hypoxia, TGFβ signaling, metabolic reprogramming, and MYC activation, while immunogenic tumors were associated with a significant immune infiltrate and upregulation of immune regulatory networks involved in acquired immune suppression. In addition, Moffitt et al. [63] performed a microarray analysis of primary and metastatic tumors, identified two tumor subtypes (“classical” subtype and a “basal-like” subtype), and two stromal subtypes (“normal” subtype and “activated” subtype). The classical subtype was associated with poor prognosis and most of the identified genes overlap with the “classical” group gene by Collison et al. [49].

The above-described genetic classifications reflect the intrinsic molecular characteristics of tumors, allowing a better definition of the clinical heterogeneity of cancers. A relationship between K-RAS dependency and some of the above-classified subtypes have been investigated [49]. Specifically, by using an RNAi-based assay to deplete *K-RAS*, it was observed that classical subtype tumors cells were more dependent on *K-RAS* than quasi-mesenchymal PDAC cell lines. Interestingly, the gene expression signature associated with K-RAS addiction validated by Singh et al. [47] suggests that genes associated with an epithelial phenotype might represent potential biomarkers of K-RAS dependency in PDAC. Kapoor et al. [50] demonstrated a significant association between K-RAS-independent tumors and cells with the quasi-mesenchymal subtype; yet the ability of these genetic classifications to act as predictive biomarkers of K-RAS dependency for translational purposes needs to be validated.

## 4. Scores of K-RAS Dependency Based on Metabolic Phenotypes Analyses

K-RAS oncogenic activation orchestrates metabolic reprogramming crucial for tumor growth, proliferation, and survival. Metabolic phenotypes associated with K-RAS might represent a fingerprint of K-RAS activation status as well as vulnerability at the metabolic level. The tumor microenvironment of the PDAC is characterized by dense desmoplastic regions that make it poorly vascularized causing a reduction in oxygen supply and reduced nutrient delivery to cells [64]. In this context, K-RAS promotes glucose uptake and enhances glycolysis by inducing the expression of GLUT1, the glucose transporter, and other key glycolysis enzymes including Hk1, Hk2, Pfk1, and LdhA [18]. In addition, in order to promote the increase of glycolysis and support tumor cell viability, K-RAS increases the hexosamine biosynthesis pathway (HBP) and the non-oxidative arm of the pentose phosphate pathway (PPP); the latter generates ribose-5-phosphate for de novo nucleotide biosynthesis [18,65]. Mitochondrial reactive oxygen species (ROS) levels are also essential for K-RAS mediated transformation and growth of PDAC cells [66,67]. Interestingly, in PDAC, low intracellular ROS levels were associated with tumorigenesis compared to other tumors in which increased ROS levels were associated with tumor progression [68,69]. A non-canonical glutamine metabolism pathway to maintain redox homeostasis and instrumental to PDAC growth has also been described [66]. Beyond glycolysis and glutamine metabolism, oncogenic K-RAS also promotes autophagy to recycle the metabolites and to maintain cell viability and survival. A study from K-RAS-driven tumor cell lines and from PDAC patients highlighted an increased expression of gene encoding protein related to autophagosome formation, and the expression of these genes correlated with a worse clinical outcome in cancer patients [70]. Indeed, the pharmacological or genetic inhibition of core components of the autophagy process impaired the growth of PDAC cell lines and PDAC development in a K-RAS-driven mouse model as well as in PDAC-PDX models [71]. Furthermore, oncogenic K-RAS promoted macropinocytosis to transport extracellular proteins as an amino acid source for the tricarboxylic acid (TCA) cycle to sustain tumor growth [72]. Although the above-mentioned studies underline metabolic phenotypes that have been associated with the activation of K-RAS, whether these pathways might represent biomarkers or vulnerability of K-RAS-dependent tumors requires further investigation.

On the other hand, other studies performed a metabolomics approach in selected K-RAS-dependent and K-RAS-independent PDAC cell lines after K-RAS depletion, in order to more specifically decipher the key metabolic signatures associated with K-RAS dependency or independency. A widely integrated transcriptomic and metabolomic analysis showed a significant gene expression profile of metabolic features of K-RAS-independent tumors [73]. In this study, a subpopulation of cells that relapsed after genetic and pharmacologic ablation of the K-RAS pathway was isolated. Further characterization of these K-RAS resistant cells by transcriptomic analysis revealed the expression of key regulators of mitochondrial functions, autophagy, and lysosome activity, while metabolomic assays revealed a decrease of metabolic intermediates involved in tricarboxylic acid, impaired glycolysis, and dependency on oxidative phosphorylation (OXHPOS) as energy source and for survival [73].

K-RAS-dependent PDAC cells showed a clear upregulation of the pyrimidine biosynthetic pathways, as demonstrated by several reports. Codina et al. showed that after treatment with MEK inhibitors, K-RAS-dependent PDAC cells exhibited MYC protein downregulation, which compromised nucleotide biosynthesis, essential to support growth and the survival of K-RAS-dependent cell lines [52]. Metabolomics analysis conducted in K-RAS-dependent and independent PDAC cell lines further validated the key role of pyrimidine biosynthesis and glutamine metabolism to characterize PDAC cells with a K-RAS addiction [52]. In a different study, a 3D clonogenic synthetic lethal screening exploiting a wide range of compounds identified dihydroorotate dehydrogenase (DHODH) inhibitors as major pharmacological suppressors for the growth of K-RAS- dependent cell line. DHODH enzyme regulates de novo pyrimidine biosynthesis and its inhibition reduced, beyond pyrimidine biosynthesis, cellular levels of glutamine and glutamate, showing an antitumor effect in mice models [74]. Mottini et al. [54] showed that the cytosine analog and U.S. Food and Drug Administration (FDA)-approved drug decitabine induced the impairment of nucleotide biosynthesis and deoxyriboNucleotide TriPhosphate (dNTP) pool homeostasis selectively in K-RAS-dependent PDAC but not in K-RAS-independent PDAC cells, showing a selective cytotoxic effect. This depended on an intimate connection between K-RAS-dependent tumor cells and the pyrimidine metabolism. Overall, these studies indicated that the upregulation of key metabolites or enzymes belonging to the pyrimidine biosynthesis can act as a metabolic biomarker to score a phenotypic dependency of PDAC cells on K-RAS (Table 2). In this context, further investigation into the expression or activity of key enzymes of the pyrimidine biosynthetic pathways as prognostic markers in PDAC is needed.

## 5. Scores of KRAS Dependency Based on Tumor Microenvironment and Immunogenicity

In the last few years, there has been growing interest in the role of the tumor microenvironment in PDAC development due to its role in tumor progression, invasiveness, and promoting therapies resistance. The pancreatic cancer microenvironment consists of cancer cells and non-neoplastic cells including pancreatic stellate cells (PSCs), regulatory T cells (Tregs), tumor-associated macrophages (TAMs), myeloid-derived suppressor cells (MDSCs), fibroblasts, and extracellular matrix components [75,76]. The crosstalk between the microenvironment and cancer cells induces the release of cytokines, chemokines, growth factors, and metalloproteases that help to recruit active TAMs and cancer-associated fibroblasts (CAFs), which support tumor progression and metastasis [77]. Notably, these dynamic and reciprocal interactions promote desmoplasia, with a low tumor perfusion microenvironment characterized by a dense desmoplastic stroma, which hinders drug delivery and suppresses antitumor immune response [78,79]. In addition, PDACs are often immunologically cold, as defined by the lack of effector T cells infiltrating the tumor. Traditionally, this phenomenon is partially attributed to the low mutational load of PDAC. In this scenario, it is worth asking to what extent mechanisms linked with K-RAS dependency or independency might play a role in the immunogenicity and the immune response of PDAC, and thus, whether these immunogenic phenotypes might act as biomarkers of tumor dependency. Various high-throughput genomic and transcriptomic analyses have revealed a key role of K-RAS signaling in promoting mechanisms of escape from immune surveillance [80]. K-RAS-mutated cancer cells secrete tumor-derived granulocyte-macrophage colony-stimulating factor (GM-CSF), promoting the recruitment and expansion of MDSCs in the microenvironment, which are able to suppress the antitumor activity of CD8 cytotoxic T cells [81]. Oncogenic K-RAS recruits myeloid cells through the secretion of cytokines including interleukin 6 (IL-6), IL-13, CCL2, Granulocyte Colony-Stimulating Factor (G-CSF), Macrophage Colony-Stimulating Factor (M-CSF), and GM-CSF [75]. Furthermore, evidence in mouse models showed that K-RAS-dependent signaling activated CAFs through the induction of Hedgehog ligands, regulated extracellular matrix (ECM) remodeling, and promoted collagen degradation by matrix metalloproteinases (MMPs), facilitating angiogenesis, tumor cells invasion, and metastasis [17,18,80,82,83]. Integrated genomic, transcriptomic, and immunological analysis on some PDAC subtypes revealed an immunosuppressive microenvironment in the quasi-mesenchymal subtype described by Collison [49] that correlated with high numbers of Tregs and M2 macrophages, and a low number of effector T cells. On the contrary, the classical subtype consisted of abundant M1 macrophages, CD4 and CD8 T cells, and a low number of Tregs and M2 macrophages, suggesting a immune responsive microenvironment [62,63]. Since classical and quasi-mesenchymal subtypes have a strong correlation with *K-RAS* dependency and independency, respectively, it would be important to further investigate if the immune-phenotypes correlate directly with *K-RAS* dependency and how K-RAS modulation affects the immunogenic phenotypes of PDAC.

## 6. Therapeutic Opportunities Against K-RAS-Dependent PDAC

Strategies developed to target K-RAS and its downstream effectors are likely to elicit a stronger therapeutic response against K-RAS-dependent tumors. Far from exhaustive, this section will provide some examples of these strategies, including direct K-RAS inhibitors, inhibitors of plasma membrane association, inhibitors of downstream signaling, and of metabolic phenotypes. The first compounds identified as capable of directly inhibiting mutant K-RAS proteins were small molecules able to interfere with the K-RAS-Guanosine Diphosphate (GDP) complex and inhibit Son of Sevenless homolog (SOS)-mediated nucleotide exchange [84,85,86,87]; other compounds instead efficiently were able to bind to RAS-GTP, thus inhibiting signaling cascades downstream of K-RAS. However, these compounds have not yet been investigated in clinical settings [88,89]. The targeting of enzymes involved in the post-translational modifications of K-RAS, necessary for protein activation, has been also investigated. Farnesylation is a post-translational modification crucial for the proper plasma membrane localization of K-RAS and downstream pathways activation. In this context, a farnesyltransferase inhibitor (FTI) termed tipifarnib was developed as a potential inhibitor of K-RAS [90]. Moreover, deltarasin, a small molecule that binds the prenyl-binding protein PDEδ, that is crucial for plasma membrane localization of farnesylated K-RAS, has also been developed [91,92]. However, clinical trials did not show a significant anti-tumor effect and any survival benefit for patients [93,94].

Current efforts to block activated K-RAS are also focused on downstream K-RAS-dependent pathways. One of the commonly studied pathways is the RAF-MEK-ERK pathway, and several MEK inhibitors have been developed including trametinib and selumetinib [95,96]. Clinical trials’ results related to these inhibitors failed to show clinical benefit and effect on survival in patients [97,98]. However, a few phase I/II studies are underway to test the efficacy of other MEK inhibitors including pimasertib and refametinib in combination with gemcitabine [96,99,100]. Several small molecules have been developed to target PI3K-, AKT-, and/or the Mammalian Target Of Rapamycin (mTOR)-dependent pathway, but monotherapies with PI3K-dependent pathway inhibitors alone failed to show efficacy in K-RAS-mutant cancers [101]. However, the combination of PI3K with RAF-MEK-ERK inhibitors exhibited potent tumor growth inhibitory activity [33,87,102], but clinical results do not match with those seen in preclinical models [103]. Importantly, in most of the clinical studies cited above, the assessment for K-RAS dependency has not been performed before treatments, thus therapies were not tailored for the patients’ population which were highly likely to respond.

Recently, preclinical evidence revealed a specific covalent inhibitor with high selectivity for K-RAS^G12C^ able to trap the inactive K-RAS-GDP complex, thus blocking nucleotide exchange and RAS downstream signaling [104]. Currently, the agent is being evaluated in a phase I/II clinical trial (NCT03600883) for patients with advanced solid tumors harboring a *K-RAS^G12C^* mutation. Nonetheless, G12C mutations are rarely observed in PDAC (1%), and similar approaches targeting K-RAS^G12D^ and K-RAS^G12V^ mutations, which constitute the prevalent K-RAS mutations in PDAC are needed [19].

Autophagy and macropinocytosis are both biological mechanisms that contribute to the growth and survival of K-RAS mutant pancreatic cancer cells [71,72], and clinical studies are evaluating hydroxychloroquine as autophagy inhibitors in combination with other chemotherapeutic drugs [105].

Finally, the dependency on pyrimidine metabolism in K-RAS-dependent PDAC has been exploited in preclinical models by Mottini et al. [54]. Thanks to a computational drug repositioning approach using K-RAS-driven signatures, authors repurposed 5-aza-2’-deoxycytidine (decitabine), an FDA-approved drug, to inhibit K-RAS-dependent PDAC tumor growth. K-RAS-dependent PDACs were highly sensitive to decitabine treatment, showing reduced cell viability and impaired tumor growth. On the contrary, decitabine treatment in K-RAS-independent cell lines and tumors did show minimal or no effect.

In conclusion, several therapeutics have been developed especially for treating K-RAS-driven PDAC and tested in preclinical or clinical settings. However, in most cases, K-RAS dependency has not been assessed on the treated population, and the response rate upon treatments has not been evaluated on the basis of the effective K-RAS dependency of tumors. Based on the emergency of biomarkers for K-RAS dependency, as described in this review, the results of clinical trials and drug effectiveness should be reevaluated for a complete assessment of drug efficacy in PDAC.

## 7. Conclusive Remarks

Mutated K-RAS is one of the most important and validated molecular antitumor targets in PDAC and the development of therapeutics against K-RAS is under active preclinical and clinical investigation. Treating tumors with an effective phenotypic dependency on K-RAS will result in an increased likelihood to observe a therapeutic response. Scoring K-RAS dependency by means of transcriptomic or metabolomics profiling has the potential to move forward to a new generation of molecular stratification of tumors for diagnostic purposes. To accomplish this, key questions need to be addressed: What are the tumor phenotypes under the active control of oncogenic K-RAS? Could such phenotypes be readily probed into the clinical routine? Another important open question is whether tumor heterogeneity, a key aspect limiting the effectiveness of targeted therapies, is relevant in the case of K-RAS dependency, and therefore if a small percentage of K-RAS-independent cells might co-exist in the bulk of dependent tumor cells, a circumstance that could potentially limit the efficacy of any targeted therapies. Single cell-based resolution methodologies will be necessary to solve such a question. Finally, it is important to understand how dependency would be also layered by stroma and other tumor microenvironment components, thus increasing the arsenal of specific K-RAS-driven molecular markers for certain PDAC subtypes.

## Figures and Tables

**Table 1:** Listing of upregulated and downregulated genes related to K-RAS pathway activation [45] and to K-RAS dependency [44].

Ref	Gene Symbol		Methodologies
	Up regulated genes	Down regulated genes	
Loboda et al., 2010	*ADAM8, ADRB2, ANGPTL4, ARNTL2,C19orf10, C20orf42, CALM2,CALU, CAPZA1, CCL20, CD274, CDCP1, CLCF1, CSNK1D, CXCL1, CXCL2,CXCL3, CXCL5, DENND2C, DUSP1, DUSP4, DUSP5, DUSP6, EFNB1, EGR1, EHD1, ELK3, EREG, FOS, FOXQ1, G0S2, GDF15, GLTP, HBEGF, IER3, IL13RA2, IL1A, IL1B, IL8, ITGA2, ITPR3, KCNK1, KCNN4, KLF5, KLF6, LAMA3, LDLR, LHFPL2, LIF, MALL, MAP1LC3B, MAST4, MMP14, MXD1, NAV3, NDRG1, NFKBIZ, NPAL1, NT5E, OXSR1, PBEF1, PHLDA1, PHLDA2, PI3, PIK3CD, PIM1, PLAUR, PNMA2, PPP1R15A, PRNP, PTGS2, PTHLH, PTPRE, PTX3, PVR, RPRC1, S100A6, SDC1, SDC4, SEMA4B, SERPINB1, SERPINB2, SERPINB5, SESN2, SFN, SLC16A3, SLC2A14, SLC2A3, SLC9A1, SPRY4, TFPI2, TGFA, TIMP1, TMEM45B, TNFRSF10A, TNFRSF10B, TNFRSF12A, TNS4, TOR1AIP1, TSC22D1, TUBA1, UAP1, UPP1, VEGF, ZFP36*	*ABCC5, ARMC8, ATPAF1, AUTS2, C1orf96, C6orf182, CELSR2, CENTB2, COQ7, DRD4, ENAH, HNRPU, HTATSF1, ID4, ITSN1, JMJD2C, KIAA1772, MIB1, MRPS14, MSI1, MSI2, NUP133, OGN, PARP1, PIAS1, RASL10B, RFPL3S, RTN3, SEC63, SF4, SH3GL2, SMAD9, STARD7, TBC1D24,TMEFF1, TTC28, TXNDC4, ZNF292, ZNF441, ZNF493, ZNF669, ZNF672*	K-RAS pathway signature derived from a superset of lung cancer, breast cancer, and colon cancer gene expression data
Singh et al., 2009	*SYK, ST14, TMEM30B, SPINT1, RAB25, C1orf172, GRHL2, GALNT3, SCNN1A, EVA1, ITGB6, C1orf74, PCDH1, C6orf141, HS3ST1, CDS1, DNAJA4, CLDN7, SCEL, SCIN, ANKRD22, MAL2, EHF, RAB17, C1orf106, TTC9, DENND1C, CEACAM6, MAPK13, LOC196264, BSPRY, C1orf116, VSIG1, KIAA0703, TMPRSS4, TGFA, EPN3, ERBB3, C1orf210, TMEM45B, RALGPS2, CDA, CDH1, SYTL5, FRK, OVOL2, RDHE2, LOC653857, B3GNT3, DPP4, PRSS22, EPS8L1, RBM35B, EFNB2, CGNL1, LAMA3, PGM2L1, ELF3, PLEKHA7, TIAF1, C11orf52, EPB41L5, KRTCAP3, RAB11FIP4, PPL, DSC2, TACSTD1, FER1L4, IRF6, TSPAN1, MAOA, CLDN4 TMEM154 MYO1D, GPR115, PPP1R14C, PKIB, TSPAN15, SH2D3A, AMPD3, UBD, MTAC2D1, TMC5, AIM1, ACP6, AREG, FAM102A, ZNF608, TMEM65, KIAA1522, C5orf4, NFATC3, KLF7, ELL2, OTUB2, PLEKHG1, FUT2, SORL1, MST1R, IKZF2, KRT7, C4orf34, JAG1, HOOK1, DLG3, KCNMB4, C12orf46, FLJ20273, RAC2, Gcom1, KIAA1107, STAP2, TACSTD2, SCARB2, CGN, PRSS8, DHRS3, C1orf34, FBP1, ZNF468, GDPD3, EGLN3, SEMA4B, ARHGEF3, LOC146795, RIPK4, RASEF, PRKCH, SLC37A1, EPPK1, PROM2, STON2, JUP, EPHB3, RPS6KA2, ALDH1A3, ROD1, PAK6, WFDC2, TMEM87B, SP110, C19orf21, TNFSF13, HPGD, ERO1L, ADAM8, ARSD, CYB561, FAM84B, FA2H, F11R, ALAD, EMG1, IL13RA1, TNFRSF21, PON3, FAM83H, GNA15, VEGF, YWHAZ, ARHGEF10L, SLC41A2, ACOT11, NR3C2, KIAA1217, GCHFR, KALRN, INPP4B, ST3GAL5, SAMD9, LMCD1, CD24, WFDC3, TMEM49, DOC1, AMDD, CTNND1, TGOLN2, MCTP2, CST6, CSPG2, CHCHD7, TMC6, TMEM125, PRRG4, GSN, DKFZP779L1068 CEACAM1, CAB39, MXD1, SHROOM3, LYPD3, LAMC2, ENTPD3, PADI1, ADAM28, TMC4, DAAM1, IL23A, SNN, SOX4, TXNIP, LLGL2, PRSS16, IDS, PTK6, CDH3, CAPN8, MTUS1, STOM, CEACAM19, S100A16, HOOK2, CDKN2A, APRIN, KLF5, DAPP1, ABLIM3, PDE5A, REPS2, LRRC1, JUNB, SLC40A1, ZNRF1, PSD4, KIAA1815, PAK1, KIF21B, SLC44A3, ELF1, F5, SPINT2, FGFBP1, TRIOBP, ROR1, ATP8B1, KRAS, IFIH1, TSGA10, FUT3, EDG4, ZBTB25, TJP2, MALAT, 1 B3GNT5, FUCA1, FOXP1, MET, GBP2, RPL41, NRP2, SHROOM2, SERPINA1, TMTC2, GRK5, UCA1, LOC58489, CEACAM5, RASD1, TSC22D3, CBR3, ARHGDIB, FRMD4B, S100A6, ZNF626, F3, EPHA1, PLS1, TAF9, RPH3A, SLC44A2, FAM83A, CNKSR1, KIAA0251, GPR110, DENND2D, BIK, KIAA0284, CAMP2, AZGP1, BMF, CHMP4C.*	*HNRPU, SLC39A14, PARVB, SH2B3, FLJ45482, NEDD4, IPO7, SGPP1, USP47 HIST1H1D,, FGFR1, MRC2, MSX1, FGF2, TEAD4, AGPAT5,, WDHD1, B4GALT6, TTC28, NFIC, RAPGEF1, ZIC2, RAB6IP1, RECK, LHFP, ST3GAL3 MSRB3, SLC26A2, PMP22, MAGEH1, BMP6, ROBO3, GJA7, TMEM20, MCOLN2, SEC61A2, IL11RA, COPZ2, NIN, ANTXR1, RSAD1, EEF2K, ITPR2, C14orf135, CWF19L1, ANKRD28, PPP4R2 TMEM118, TSPAN4, RAGE, DYRK4, FLJ36166, ALPK2, BCAP29, C14orf139, CSPG5, TTC7B SATB2, TCF8, SLC35B4, OSTM1, IKIP, SFXN1, TRIM7, KIAA1212, MGC39900, NFIX, PDLIM3, MIB1, MLSTD2, LOC401068, ALS2CR4, PRG1, APLN, FAM101B, LOC541471, HNRPA2B1, RHOT1, LOC153346, DYRK3, EML1, RYK KCTD15, PAX6, PLCB4, WDR35, CHRNA7, LIX1L, ACTA2, HTRA1, ABP1, ANXA6, HSPA12A, MAGEE1, SYDE1, TUB, SMARCD3, NUDT11, SYNGR1, MPHOSPH9, ADRA2C, TXNRD1, EPB41L5, MPPE1, SLC1A3, LOC439949, FLJ10847*	K-RAS dependency signature derived from a subset of K-RAS dependent primary lung tumors of squamous carcinoma and adenocarcinoma subtypes

**Table 2:** Comparison of previously published metabolic profiles of K-RAS-dependent and independent pancreatic cancer cell lines and tumors.

Ref	Methodologies	Pathways/Metabolites Analyzed
Santana Codina N et al., 2018	LC-MS/MS analyisis in K-RAS sensitive and resistant cells	pentose phosphate pathway (PPP) and nucleotide biosynthesis and glycolysis
Mottini C et al., 2019	LC/MS analysis from both dependent and independent PDAC cell lines	nucleotide metabolism and pyrimidine biosynthesis
Koundinya M et al., 2018	Mass spectrometric analysis for K-RAS dependent and independent cells and tumor tissues	de novo pyrimidine biosynthetic pathway
Viale A et al., 2014	metabolomic analysis using a LC-MS/MS in a subpopulation of dormant tumor cells surviving K-RAS ablation	Tricarboxylic acid cycle (TCA) intermediates, nucleotide triphosphates, deoxynucleotide triphosphates, glutathione (GSH) and glutathione disulphide levels
